# Evidence for the Involvement of the Chemosensory Protein AgosCSP5 in Resistance to Insecticides in the Cotton Aphid, *Aphis gossypii*

**DOI:** 10.3390/insects12040335

**Published:** 2021-04-09

**Authors:** Fen Li, Herbert Venthur, Shang Wang, Rafael A. Homem, Jing-Jiang Zhou

**Affiliations:** 1 Department of Plant Protection, Hainan University, Haikou 570228, China; lifen2010happy@sina.com; 2 Laboratorio de Química Ecológica, Departamento de Ciencias Químicas y Recursos Naturales, Centro de Investigación Biotecnológica, Aplicada al Medio Ambiente (CIBAMA), Universidad de La Frontera, Temuco 54-D, Chile; herbert.venthur@ufrontera.cl; 3 College of Plant Sciences, Jilin University, Changchun 130062, China; wangshang15@mails.jlu.edu.cn; 4 Department of Biointeractions and Crop Protection, Rothamsted Research, Harpenden AL5 2JQ, UK; rahomem@gmail.com; 5 Biocontrol Engineering Laboratory of Crop Diseases and Pests of Gansu Province, Gansu Agricultural University, No. 1 Yingmen Village, Anning District, Lanzhou 730070, China; 6 State Key Laboratory of Green Pesticide and Agricultural Bioengineering, Ministry of Education, Guizhou University, Huaxi District, Guiyang 550025, China

**Keywords:** chemosensory protein, pesticide resistance, insect pest management

## Abstract

**Simple Summary:**

Insect chemosensory proteins (CSPs) are potential targets for insect pest control strategies and are proposed to function in insect chemoreception, because they play a role in crop host location by binding and transporting odorant molecules. They are also thought to have other functions, for example, in tissue regeneration and in insecticide resistance, because they also express in nonolfactory tissues and are capable of binding insecticides. However, there are few reports that provide direct evidence for this proposal. In this study, we discovered gene gain-and-loss among aphid populations, possibly associated with different insecticide resistance, and then identified and cloned a CSP gene responsive to insecticide treatments. The introduction of such gene in *Drosophila* fruit flies made the transgenic flies less sensitive to the treatment of different insecticides. Our study advances the research of insect CSP functions and offers valuable new information to target CSPs for pest management.

**Abstract:**

It has been speculated that insect chemosensory proteins (CSPs) may have additional roles beyond olfaction. In this study, the phylogenetic and genomic analyses of the CSPs of the cotton aphid, *Aphis gossypii*, revealed the presence of gene gain-and-loss among different aphid field populations. Differential expressions of eight *CSP* genes were demonstrated after treatments with insecticides of different modes of action. The expression of *AgosCSP5* was significantly upregulated by the insecticide treatments in a dose-dependent manner. The *Drosophila* flies overexpressing *AgosCSP5* were significantly less susceptible to the insecticides, omethoate, imidacloprid and cypermethrin but not to deltamethrin and tau-fluvalinate, compared with control flies. The transgenic *Drosophila* flies exhibited an LC_50_ resistance ratio of 2.6 to omethoate, compared with control flies. Likewise, the mortality of the transgenic flies to imidacloprid and cypermethrin was significantly lower than that of the control flies (*p* < 0.01). Homology modelling, molecular docking and dynamic simulation supported the interactions and revealed a higher stability of AgosCSP5/insecticide complexes than AgosCSP5/semiochemical complexes. Our study demonstrates for first time the in vivo evidence for the involvement of *CSP* genes in insecticide resistance of crop insect pests and provides new insights of the newly discovered CSP-mediated insect resistance mechanism to insecticides.

## 1. Introduction

Control of insect pests of commercial field crops is still reliant on a considerable extent on the use of chemical insecticides, which act primarily on several targets in the central nervous system (CNS), i.e., acetylcholinesterase, sodium channels, chloride ion channels, acetylcholine receptor, ryanodine receptors, mineralocorticoid receptors and octopamine receptors [1,2]. However, the development of resistance to insecticides has been widely reported in crop and public health pests and can be a significant issue in some cases. The most commonly reported resistance mechanisms to insecticides involve altered target-site and metabolic resistance [2], as well as behavioural resistance and reduced cuticular penetration [3,4]. Recently, a new insecticide resistance mechanism to pyrethroid insecticides mediated by the mosquito chemosensory protein (CSP) AgamSAP2 was reported [5,6].

CSPs are one of the small and water-soluble proteins that are thought to be involved in insect chemosensory perception at the peripheral nerve system by binding and transporting odorant molecules to chemosensory/olfactory receptors (ORs) on olfactory neurons in insect antennae. ORs then convert the chemical signals into physiological electrical signals or nerve impulses [7,8]. The signal transduction continues along nerve axon to the CNS and finally triggers specific behaviours [7]. CSPs are found not only in insects but also in noninsect arthropods, such as the brine shrimp *Artemia franciscana* [9,10]. It has been reported that CSPs have a wide binding spectrum and are broadly expressed in various nonolfactory tissues [9,11]. 

Nine CSP genes have been identified in the cotton aphid *Aphis gossypii* Glover (Hemiptera: Aphididae) [12,13]. However, their precise physiological and biochemical functions have not been specifically studied. Numerus studies suggest that insect CSPs are involved in the physiological processes beyond insect chemoreception [1,5,6,8,10,11]. The upregulation of CSP gene expression has been associated with insecticide exposure [14,15,16,17]. Lin et al. (2018) [18] showed the direct binding between CSPs of *Spodoptera litura* and insecticides (chlorpyrifos, emamectin benzoate, fipronil) using fluorescence competitive binding assay. Subsequent RT-qPCR and RNAi experiments suggested that SlituCSP18 might mediate the insecticide resistance [19]. Li et al. (2017) [20] showed that imidacloprid, at the sublethal dose, significantly inhibited the binding ability of *Apis cerana* CSP1 (AcerCSP1) to the natural ligand, β-ionone, in fluorescence competitive binding assay. These studies indicate that CSPs could play a role in insecticide resistance. Only last year, in 2020, was one CSP shown to confer the resistance to the pyrethroid insecticide deltamethrin in the malaria mosquito Anopheles gambiae [6]. 

The cotton aphid is one of the most economically important agriculture pests worldwide, and the polyphagous aphid species [21] and has evolved strong pesticide resistance to virous insecticides [22,23,24]. The aim of this study is to assess whether CSPs are associated with insecticide resistance in the cotton aphid *A. gossypii*. Firstly, we annotated all *CSP* genes from the genomic and several transcriptomic datasets of different field populations. We then examined the expression profiles of these *AgosCSP* genes after insecticide treatments and functionally validated the role of *AgosCSP5* in conferring insecticide resistance in the transgenic *Drosophila* flies overexpressing this gene. Lastly, we performed ligand docking and molecular dynamics analyses to examine the affinity and stability of the interactions between AgosCSP5 and insecticides with different modes of action.

## 2. Materials and Methods

### 2.1. Insect

The cotton aphids were originally colonized from a single aphid collected in the cotton field of Yuncheng, Shanxi Province, China, and reared on the cotton seeding *Gossypium hirsutum* (Linn.) at standard environmental conditions of 22 ± 1 °C, 70 ± 10% relative humidity and a photoperiod of 16 h:8 h (light:dark) in a climate chamber. These cotton aphids have been exposed to different insecticides and assumed to have developed resistance to the insecticides used in this study.

The fruit flies *Drosophila melanogaster* [*y w M(eGFP, vas-int, dmRFP)ZH-2A; P{CaryP}attP40*] were sourced from Cambridge fly facility and used to examine their susceptibility to insecticides. The transgenic flies were generated by integrating into the *Drosophila* genome the pUAST plasmid containing an aphid CSP gene at attP40 site in chromosome 2. The control flies had exactly the same genotype with an empty pUAST plasmid integrated at the same attP40 site. 

### 2.2. Insecticides 

The insecticides used include an organophosphate insecticide omethoate (an acetylcholinesterase inhibitor); a neonicotinoid insecticide imidacloprid (a nicotinic acetylcholine receptor blocker); and three pyrethroid insecticides, cypermethrin (a sodium channel blocker), deltamethrin (contact and ingestion toxic) and tau-fluvalinate (sodium channel permeability modifier). All insecticides were purchased from Sigma–Aldrich with more than 95% purity.

### 2.3. Identification and Bioinformatics Analysis of AgosCSP Genes 

The *A*. *gossypii CSP* genes (*AgosCSP*) were identified by Basic Local Alignment Search Tool (BLAST) searching [25], using the sequences of previously identified *AgosCSPs* [23] and the pea aphid *CSP* genes [26], against the transcriptome assembly [27] and the cotton aphid genome (*A. gossypii* genome v1.0) on the Aphidbase (https://bipaa.genouest.org/sp/aphis_gossypii/blast/) as on 6th April 2021 with the default parameters.

**Figure 1:**
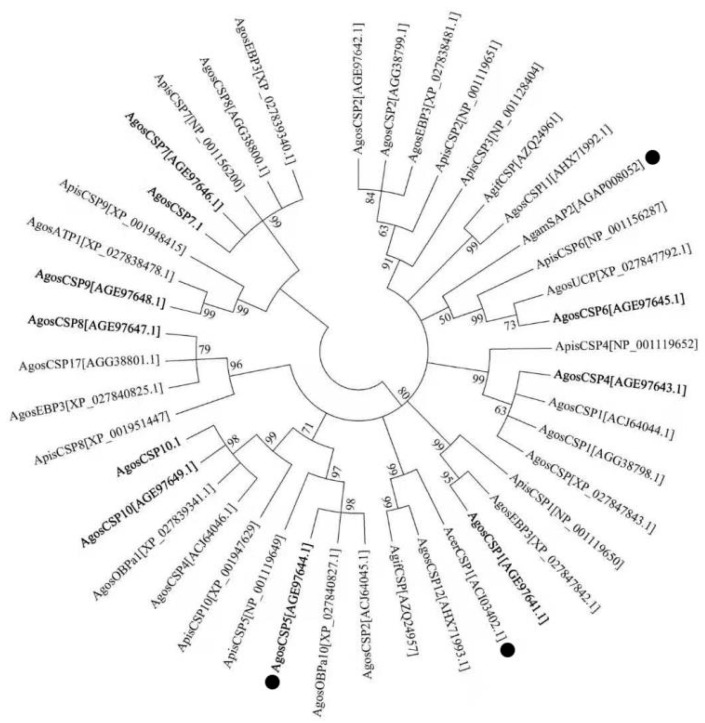
Phylogenetic tree for aphid chemosensory proteins (CSPs) constructed using the maximum likelihood method with 1000 replicates. The bootstrapping values of replicate trees with the associated clustering are shown at each node. The CSPs that were identified in the cotton aphid population used by the current study are in bold. AgosCSP2 and AgosCSP3 were not identified from this cotton aphid population. CSPs identified from five cotton aphid populations (this study; Gu et al., 2013 [12]; Xu et al., 2009 [13]; Li et al., 2013 [deposition in the National Center for Biotechnology Information (NCBI)]; and Quan et al., 2019 [29]) were used. The CSPs that have been shown to involve in insecticide binding and resistance, AgamSAP2, AcerCSP1 and AgosCSP5 are indicated by black dot. Agos: *A. gossypii*, Apis: *A. pisum*, Acer: *Apis cerana*, Agif: *Aphidius gifuensis*; Agam: *Anopheles gambiae*. CSP: chemosensory protein; ATP1: allergen Tha p 1-like; EBP3: ejaculatory bulb-specific protein 3-like; OBPa10: odorant-binding protein A10. UCP: uncharacterised protein.

Alignment of CSP proteins was performed with ClustalX 2.1 and loaded to MEGA 6.0 [28] to construct the phylogenetic tree using the maximum likelihood method with 1000 bootstrap replicates. The accession numbers of all CSPs used are given in Figure 1. 

### 2.4. Generation of Transgenic Drosophila Flies Expressing AgosCSP5

*Drosophila* strains used and produced in the current study were maintained on the standard Bloomington food at 24 °C and 65% RH under a 12/12-h light/dark cycle. The sequence of *AgosCSP5* (KC161567.1) was codon optimised for *D. melanogaster* expression (GeneArt^TM^–ThermoFisher Scientific) and cloned into the *pUASTattB* plasmid (GenBank: EF362409.1). The *pUASTattB–AgosCSP5* construct was microinjected into the preblastodermal embryos of an integration strain [*y w M(eGFP, vas-int, dmRFP)ZH-2A; P{CaryP}attP40*] under an inverted microscope (eclipse TieU Nikon, Japan) equipped with a 10 × /0.25 lens, 10 × /22 eyepiece and fluorescence illumination. The empty *pUASTattB* plasmid was also injected and used as control. The injection mix was comprised of 0.5 × phosphate buffer (pH 6.8, 0.05 mM sodium phosphate, 2.5 mM potassium chloride), 300 ng/µL of the construct and 200 mg(a.i.)/L fluorescein sodium salt and was delivered by a FemtoJet express micro injector (Eppendorf, Hamburg, Germany). Injection needles were prepared according to Miller et al. (2002) [30].

The survivors were backcrossed, and the F1 progenies were screened for the white marker gene (orange eye phenotype). Positive flies were intercrossed to generate homozygous flies (red eye phenotype) of the final strain (*UAS*–*CSP5* strain) and the control strain (*UAS*–empty strain).

### 2.5. UAS/GAL4 Expression of AgosCSP5

The *UAS–CSP5* male flies were crossed to virgin females of the heat shock inducible driver strain *Hsp70–GAL4* (w[*];P{wmC] = GAL4–Hsp70.PB}89-2-1). Progeny (F1) flies (*Hsp70–GAL4 > UAS–CSP5*) were treated with a heat shock for 30 min at 37 °C, followed by 1 h rest under 24 °C, to induce the expression of *AgosCSP5*. The flies were then treated with insecticides either immediately or 24 h after the heat shock treatment. Genomic DNA (gDNA) was isolated using phenol/chloroform extraction method with proteinase K treatment and used to confirm the presence of the transgene *AgosCSP5* in the *Drosophila* genome. 

### 2.6. Bioassays of A. Gossypii

The omethoate stock (1.0 × 10^4^ mg(a.i.)/L in methanol) was diluted to a serial of concentrations with distilled water containing (*v*/*v*) 0.05% Triton X-100 and 1% acetone. Triton X-100 was used as a surfactant to reduce surface tension and to aid spread of omethoate on cotton leaves. Omethoate toxicity was determined with the leaf-dipping method [23] by exposing apterous *A. gossypii* adults to the leaves treated with omethoate at the concentrations of 0 mg(a.i.)/L (as control), 400 mg(a.i.)/L, 800 mg(a.i.)/L, 1200 mg(a.i.)/L, 1600 mg(a.i.)/L and 2000 mg(a.i.)/L. Each concentration was replicated at least three times with at least 30 aphids. The mortality was assessed at 24 h after the treatments. Adults that did not exhibit repetitive movement of more than one leg (i.e., nonreflex) (after gentle prodding if necessary) were scored as dead [23]. LC_10_, LC_50_ and LC_90_ values were calculated using the PoloPlus 2.00 software (LeOra Software Inc., Petaluma, CA, USA). 

### 2.7. Bioassays of Drosophila Flies

Transgenic female flies at 2–5 days post-eclosion were subjected to the contact/feeding bioassay. A 5-fold serial dilution was prepared with 5000 mg(a.i.)/L omethoate stock to a concentration range from 10,000 mg(a.i.)/L to 0 mg(a.i.)/L. A total of 100 µL of each dilution was added to the surface of a settled 3 mL agar (2% *w*/*v*) containing sucrose (1.2% *w*/*v*) and acetic acid (0.4% *v*/*v*) in a *Drosophila* vial (25 mm × 95 mm). Transgenic flies were transferred to the vials. The mortality was scored after 24 h. Dead flies, as well as those displaying no coordinated movement (difficulty to walk up the vial and to lift their feet), were cumulatively scored as “dead.” At least three replicates of 20 flies per replicate were used for each concentration. LC_50_ values were calculated using PoloPlus 2.00 software. Other insecticide bioassays were performed in a similar way as described above. The insecticides used include omethoate, imidacloprid, cypermethrin, deltamethrin and tau-fluvalinate. 

### 2.8. RNA Extraction, cDNA Synthesis, RT-PCR and RT-qPCR

RNA was extracted from the insecticide treated aphids and the heat shock treated transgenic flies using RNA–Solv reagent (R6830-02, Omega Bio-tech). cDNA was synthesized with HiScript^®^ II Q RT SuperMix for qPCR with gDNA wiper (R223-01, Vazyme, Nanjing China) and used in RT-qPCR with the ChamQTM SYBR^®^ qPCR Master Mix (Vazyme, Nanjing, China). A *Drosophila* strain carrying an empty pUAST plasmid integrated at the same genomic location (hereafter referred as *UAS–empty*) was used as control. The housekeeping genes *Rpl32* (*ribosomal protein L32*) and *EF1-α* (*elongation factor 1 alpha*) (EU019874.1) were used for expression normalization. The primers were designed using Primer 5 software and listed in Supplementary Table S1. Standard curves were created based on a five-fold dilution series of cDNA (1:5, 1:25, 1:125, 1:625, 1:3125 and 1:15625) to check the primer efficiency and specificity. The corresponding RT-qPCR efficiencies (*E*) were calculated according to the equation E = (10[−1/slope] − 1 × 100) [31]. All primers used have amplification efficiencies of more than 95% and a single melting peak. The RT-qPCR reaction mix contained 10.0 μL of DNA Polymerase, 1.0 μL of both forward and reverse primers (10 μM/L) and 1.0 μL of the cDNA template (1 μg/μL) in a total volume of 20 μL. The RT-qPCR reaction was performed with ABI7500 (Applied Biosystems, Shanghai, China) under the following conditions: 95 °C for 30 s, followed by 40 cycles of 95 °C for 10 s, 60 °C for 30 s. The gene expression at mRNA level was calculated using the 2^−ΔΔCt^ method [32].

The data were statistically analysed with one-way ANOVA with Tukey’s multiple comparison test using GraphPad Prism 5 software (San Diego, CA, USA). Data are expressed as Mean ± SE from three biological replicates.

### 2.9. Protein Structure Prediction and Refinement

A homology model for AgosCSP5 with the highest C-score, known as the significance of threading template alignments and convergence of structure assembly simulations, was obtained through a threading approach for alignments using I-TASSER server (https://zhanglab.ccmb.med.umich.edu/I-TASSER/) as on 6 April 2021 [33]. 

Refinement of the AgosCSP5 model was based on molecular dynamics through NAMD v2.9 Software and CHARMM36 force field [34]. Briefly, the AgosCSP5 model was solvated with water (TIP3P model) in a cubic box, neutralized by adding Na^+^ or Cl^−^ randomly, and simulated under default periodic boundary conditions. Alpha carbons (Cα) of secondary structures were fixed with a constant force of 4.184 kJ/mol/Å. A first energy minimization of 50,000 steps was performed and followed by long simulations at 300 K and 1 bar pressure in the NTP (referred to a constant number of particles, temperature and pressure) during 50 ns. The root-mean square deviation (RMSD) trajectory was used to evaluate stability followed by stereochemical quality via ProCheck every 50 frames (i.e., low conformation energy) (Supplementary Figure S1).

### 2.10. Molecular Docking and Complex Molecular Dynamics

Insecticides (omethoate, imidacloprid, cypermethrin, tau-fluvalinate and deltamethrin) and reported semiochemicals for aphids ((*E*)-β-farnesene, (1*R*,4*E*,9*S*)-caryophyllene and (*Z*)-3-hexenyl acetate) were used as ligands for molecular docking using AutoDock Vina in rigid conformations [35]. Energy minimization for the 8 ligands was performed using MM2 minimization methods in the Chem3D 16.0 Software (Perkin Elmer). Polar hydrogens were added to the AgosCSP5 model, as well as torsional bonds for ligands. Thus, a grid box with 20 × 20 × 20 points and a default space of 1 Å was prepared via AutoGrid based on CASTp calculation server (http://sts.bioe.uic.edu/castp/calculation.html) as on 6 April 2021. For every docking run, an exhaustiveness of 500 was considered, and the best binding modes were selected (lowest free binding energy in kJ/mol).

Molecular dynamics simulations were performed for the 8 ligands bound to AgosCSP5 in a flexible system, according to the methodology reported by Venthur et al. (2019) [36]. Ligand topologies were obtained through the ACPYPE server in Bio2byte suite (https://bio2byte.be/) as on 6 April 2021. The AgosCSP5 model was solvated, neutralized and fixed, following the same protocols previously described in the VMD software. This time, long simulations were performed during 20 ns for each protein–ligand complex. The RMSD trajectory tool was used to estimate stability.

## 3. Results 

### 3.1. Sequence Annotation and Phylogenetic Analysis of A. Gossypii CSPs

A total of eight cotton aphid *CSP* genes were identified from the transcriptome analysis dataset of the cotton aphid population used in this study (unpublished), numbered as those of the pea aphid from the first aphid CSP genome annotation, based on their similarity with pea aphid CSPs [26]. These *AgosCSP* genes were then compared to the previously identified *CSP* genes [12,13,27] and those annotated in the cotton aphid genome [29]. This unifies different naming systems from different studies and cotton aphid populations from different geographical regions and, thus, allows a better comparison between homologues (Table 1). 

There is a differential expression among *AgosCSP* genes in different aphid populations (Table 1). Despite the best effort with different pairs of primers and under different PCR conditions, *AgosCSP2* and *AgosCSP3* could not be amplified by PCR from the population used in this study from the cotton field at Yuncheng, Shanxi Province, China. However, *AgosCSP2* was found in the population from the cotton field at Langfang Experimental Station, Hebei Province [24]. An identical gene with a different annotation, *AgosCSP2* (AGG38799.1), was found in the National Center for Biotechnology Information (NCBI) database (Li, 2013, unpublished), suggesting *AgosCSP2* is also present in another cotton aphid populations. *AgosCSP2* genes were also found in other aphid species [12]. In contrast, *AgosCSP3* was also not identified in the population from the cotton fields at Langfang Experimental Station, Hebei Province [24], and in Xinjiang Province, China [27]. Furthermore, *AgosCSP3* was not found in other aphid species, such as *Aphis fabae*, *Rhopalosiphum padi*, *Tuberolachnus saligun* and *Myzus persicae*, by PCR with the primers designed from *ApisCSP3* [12]. However, *AgosCSP3* is present in the cotton aphid population (*A. gossypii* isolate AGOS-L3) used for the genome sequencing project [29]. *AgosCSP2*, AgosCSP2 (AGG38799.1), ApisCSP2 and ApisCSP3 are clustered together with 89% bootstrapping support (Figure 1). Mature ApisCSP2 and ApisCSP3 have a 29.7% amino acid identity and are at same genomic location (Table 1). These results suggest that *ApisCSP2* and *ApisCSP3* might be duplicated genes from same ancestral gene. However, *ApisCSP3* has been lost in the cotton aphid and other aphid species. Furthermore, two unpublished *AgosCSPs* found in the NCBI database, *AgosCSP11* (AHX71992.1) and *AgosCSP12* (AHX71993.1), were not identified from our transcriptomes [12,27] nor in the cotton aphid population (*A. gossypii* isolate AGOS-L3) used for the genome sequencing project [29] (Table 1). Interestingly, their homologues were found in other insects, such as the eastern honeybee *Apis cerana* (*AcerCSP1*) [37], the parasitoid *Aphidius gifuensis* (*AgifCSP*) [38] and the Asian hornet *Vespa velutina* [39] (Figure 1). However, without genome sequencing, it is not possible to definitely exclude the limited transcript abundance of the undetected *CSP* genes by PCR in different aphid populations. 

There are four genomic clusters of *AgosCSPs*: *CSP4–CSP1–CSP6*, *CSP2–CSP9*, *CSP8–CSP5* and *CSP10–CSP7*. *AgosCSP7* and *AgosCSP10* have two introns, and all other eight *AgosCSP*s have one intron. Interestingly, *AgosCSP2* and *AgosCSP9* are very close, with only 248 bp insertion between them on the genomic scaffold *AgSCF0976* (Table 1). *AgosCSP6* and *AgosCSP4* are in the same genomic cluster. AgosCSP6 and AgosCSP4 (AHX71993.1) are closely clustered with the insecticide-resistance-associated CSPs, AgamSAP2 [6] and AcerCSP1 [20], with bootstrapping values of more than 50% and 99%, respectively (Figure 1).

### 3.2. Upregulation of A. Gossypii CSP Gene Expression by Insecticide Omethoate

**Figure 2:**
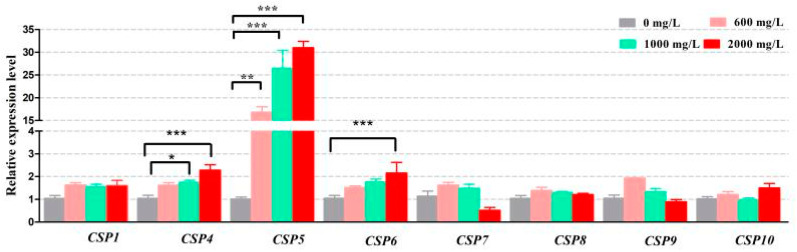
Effects of insecticide omethoate at different doses on *CSPs* expression in the cotton aphid. The results are expressed as Mean ± SEM from triplicate experiments. *, ** and *** represent *p* < 0.01, *p* < 0.001 and *p* < 0.0001 significant differences. The data were analysed using one-way ANOVA of Tukey’s multiple comparison test.

To further examine possible involvement of aphid *CSP* genes in the adaptation to environmental conditions, such as insecticide treatments, the expression levels of *AgosCSP* genes were characterized in the cotton aphids treated with omethoate at the concentrations close to LC_10_ (574.4 mg(a.i.)/L), LC_50_ (1029.1 mg(a.i.)/L) and LC_90_ (1843.8 mg(a.i.)/L) (Supplementary Table S2). The results revealed that the expression of *AgosCSP5* was significantly upregulated in a dose-dependent manner by the insecticide treatment (Figure 2) and 16.7-fold higher (*p* < 0.001) and 30.7-fold higher (*p* < 0.0001) in the aphids treated at 600 mg(a.i.)/L and 2000 mg(a.i.)/L, respectively, than that of untreated control aphids. However, the expression levels of other *CSPs* did not show such obvious induction, except for the expressions of *AgosCSP4* and *AgosCSP6* (Figure 2).

### 3.3. Expression of AgosCSP5 in Transgenic D. Melanogaster

**Figure 3:**
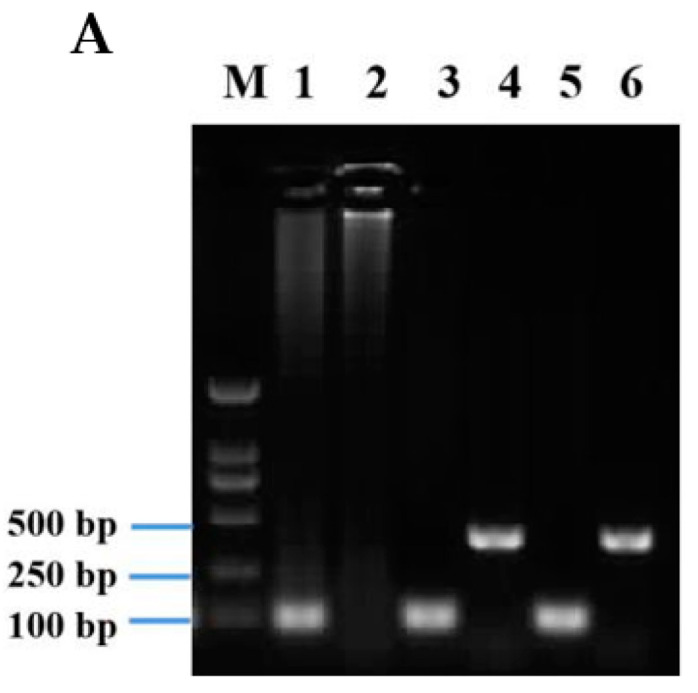

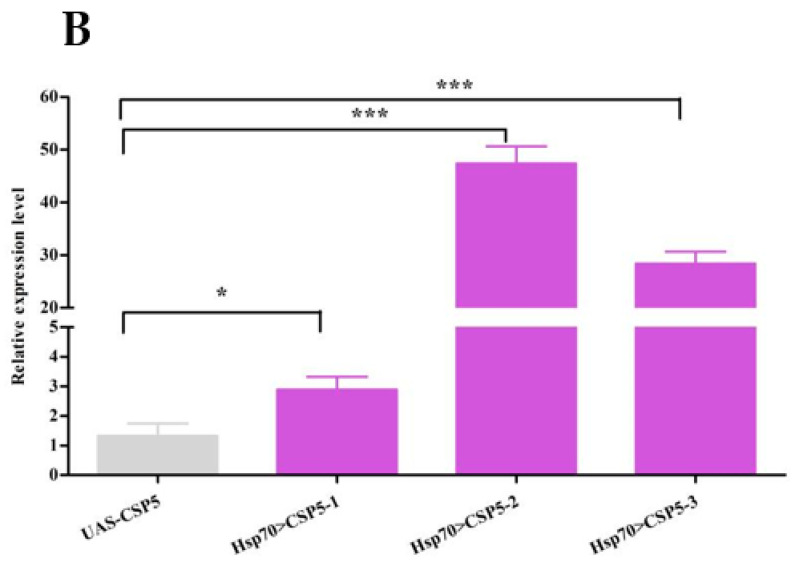
Integration of *AgosCSP5* into the genome of the transgenic *D. melanogaster.* **A**. PCR products amplified from genomic template of control *UAS–empty* flies with *Rpl32* primers (Lane 1); control *UAS–empty* flies with *CSP5* primers (Lane 2); *UAS*–*CSP5* female flies with *Rpl32* primers (Lane 3); *UAS*–*CSP5* female flies with *CSP5* primers (Lane 4); *UAS*–*CSP5* male flies with *Rpl32* primers (Lane 5); *UAS*–*CSP5* male flies with *CSP5* primers (Lane 6). M: DNA Maker. **B**. The expression levels of *AgosCSP5* gene in the transgenic flies driven by the heat shock gene Hsp70 promoter. The flies were treated with a heat shock for 30 min at 37 °C, followed by 1 h rest under 24 °C, to induce the expression of *AgosCSP5*. *UAS–CSP5*: Control parental male flies; *Hsp70 > CSP5-1:* female flies of *Hsp70*–*GAL4* x *UAS*–*CSP5* without heat shock*; Hsp70*–*CSP5*-2: female flies of *Hsp70*–*GAL4* x *UAS*–*CSP5* assessed immediately after heat shock treatment; *Hsp70*–*CSP5*-3: female flies of *Hsp70*–*GAL4* x *UAS*–*CSP5* assessed at 24 h after heat shock treatment. * and *** represent *p* < 0.01, *p* < 0.001 and *p* < 0.0001 significant differences.

In order to examine specifically the effect of the upregulation of *AgosCSP5* expression on insect susceptibility to insecticides with different modes of action, *AgosCSP5* was introduced into the fruit fly *D. melanogaster* using the GAL4/UAS system. As shown in Figure 3, *AgosCSP5* gene was confirmed to be present in the genome of the transgenic *Drosophila* flies (hereafter referred to as *UAS–CSP5* strain) (Figure 3A). These *UAS–CSP5* strains were then crossed with the *GAL4–Hsp70* strain carrying the promoter sequence of the *heat shock protein 70* (*HSP70*) gene to drive *AgosCSP5* expression under heat shock treatment in the resulting transgenic flies (*Hsp70–GAL4 > UAS–CSP5*).

After the heat shock treatment (30 min heat shock and 1 h rest), the expression level of *AgosCSP5* was upregulated by 35.6-fold and 21.4-fold higher in the *AgosCSP5*-overexpressing transgenic flies (*Hsp70–GAL4 > UAS–CSP5*) than in the control strains (*UAS–CSP5*) assessed immediately (*Hsp70*–*CSP5-*2) and at 24 h (*Hsp70*–*CSP5-*3), respectively (Figure 3B). These results demonstrate that *AgosCSP5* gene was successfully integrated into the genome of the transgenic *Drosophila* flies (*Hsp70–GAL4 > UAS–CSP5*), which were used to examine the involvement of *AgosCSP5* in the susceptibility of the transgenic flies to insecticide treatments. 

### 3.4. AgosCSP5 Confers the Resistance to Insecticides in Transgenic Drosophila Flies

**Figure 4:**
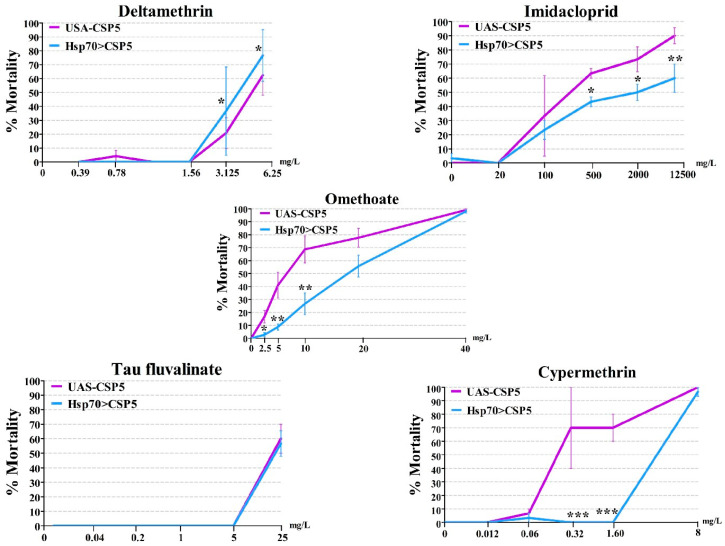
*AgosCSP5* reduction of the susceptibility of the transgenic *Drosophila* flies to insecticides. The mortalities were compared between the transgenic line (*Hsp70*–*GAL4* > *UAS*–*CSP5* with heat shock) and control line (*Hsp70*–*GAL4* > *UAS*–empty with heat shock) exposed to different doses of insecticides. *, ** and *** represent *p* < 0.01, *p* < 0.001 and *p* < 0.0001 significant differences.

The heat shock treated transgenic flies overexpressing *AgosCSP5* (*Hsp70–GAL4 > UAS–CSP5*) exhibited a higher level of resistance to omethoate (LC_50_ of 15.02 mg(a.i.)/L), compared to control flies (LC_50_ of 5.76 mg(a.i.)/L), i.e., 2.61-fold increase (Table 2). In spite of our best effort, we were unable to obtain reliable LC_50_ values of the transgenic flies treated with imidacloprid, cypermethrin, deltamethrin and tau-fluvalinate, due to irregular mortality response. However, the *Hsp70–GAL4 > UAS–CSP5* flies had significantly lower mortality than the control flies at the omethoate doses of 2.5 mg(a.i.)/L, 5 mg(a.i.)/L and 10 mg(a.i.)/L; at the cypermethrin doses of 0.32 mg(a.i.)/L and 1.60 mg(a.i.)/L; and at the imidacloprid doses of 500 mg(a.i.)/L, 2000 mg(a.i.)/L and 12,500 mg(a.i.)/L (Figure 4). The mortalities of the transgenic flies were similar to those of control flies, when tested against two other pyrethroid insecticides, deltamethrin and tau-fluvalinate.

### 3.5. AgosCSP5 Can Bind to Insecticides

We then carried out molecular locking and dynamic simulation to investigate the interaction between insecticides and AgosCSP5. For this, a homology AgosCSP5 structure model was predicted and built. The predicted AgosCSP5 structure model suggests a globular structure with seven α-helices and two disulphide bridges of typical insect CSP structures. The predicted binding site has 295.744 Å^2^ of area and 105.443 Å^3^ of volume, suggesting a big surface for ligand binding, though not deep enough, considering its volume (Supplementary Figure S2). 

Molecular docking results suggest that imidacloprid is the strongest ligand, with a free binding energy of −22.6 kJ/mol and a hydrogen bond between the hydrogen from amide sidechain of Asp19 and one of the nitrogen-bound oxygens of imidacloprid. Likewise, the binding energy scores of omethoate, deltamethrin and cypermethrin are around −16.7 kJ/mol, with the exception of −13.0 kJ/mol of tau-fluvalinate (Table 3). These binding energies are lower than those of semiochemicals, such as caryophyllene (−16.3 kJ/mol), and comparable to that of (*Z*)-3-hexenyl acetate (−18.4 kJ/mol) and higher than that of (*E*)-β-farnesene (−21.3 kJ/mol). Overall, insecticides and semiochemicals adopt similar binding modes in the binding pocket, with residues from α-helices 1, 2 and 4.

**Figure 5:**
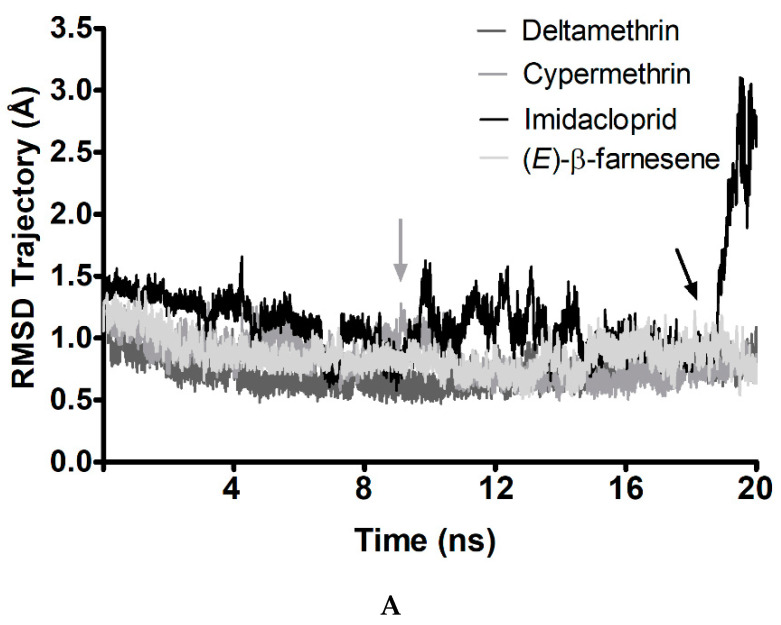

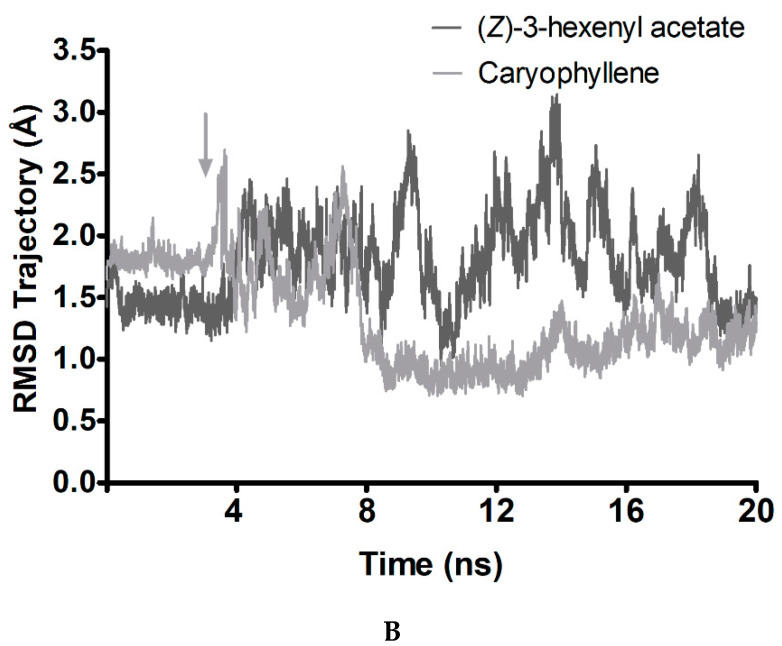
Root–mean square deviation (RMSD) trajectory (Å) from complex molecular dynamics with 20 ns of simulations for insecticides and semiochemicals bound to AgosCSP5. (**A**) RMSD trajectory for insecticides and (*E*)-β-farnesene. (**B**) RMSD trajectory for plant volatiles (*Z*)-3-hexenyl acetate and caryophyllene. The arrows indicate breakpoints in the protein–ligand trajectory. The arrows indicate breakpoints in the protein–ligand trajectory.

As the molecular docking protocol considers protein and ligands as rigid conformations (limited to some torsional bonds for ligands), molecular dynamics simulation was employed in flexible systems to test the stability of the AgosCSP5/ligand complexes. The results suggest that insecticides can retain their docked conformations in AgosCSP5 longer than semiochemicals (Figure 5). The AgosCSP5–ligand complexes with cypermethrin, deltamethrin and tau-fluvalinate were stable during the entire simulation period of 20 ns, whereas imidacloprid and omethoate only lost their bound state at the end of simulation period, but were less fluctuant than caryophyllene and (Z)-3-hexenyl acetate (Figure 5A,B). However, caryophyllene and (*Z*)-3-hexenyl acetate lost their bound state before 4 ns of simulation. Interestingly, (*E*)-β-farnesene was the only semiochemical able to stabilize in a bound conformation with AgosCSP5 (Figure 5A).

## 4. Discussion

The cotton aphid *A. gossypii* is well-known to evolve/specialize into various biotypes in regard to its life history and host plants, with various resistance mechanisms to adapt to specific natural ecological environment for its survival [23]. The current study revealed that there are *CSP* gene losses and differential expression among cotton aphid populations from different geographical regions and between aphid species (Figure 1 and Table 1). However, whether these observations underlay possible involvement of CSPs in the environmental adaptation including insecticide resistance is not clear without further study of the population genetics of these aphid populations. However, it was reported that the mosquito resistance to pyrethroid insecticides is linked to an insecticide resistance haplogroup of the CSP gene AgamSAP2 [5,6]. 

The possible involvement of insect pest CSPs in the resistance to insecticides has been indicated by in vitro insecticide binding studies [6,20] and recent study on the malaria-transmitting mosquito *A. gambiae* to the pyrethroid insecticide deltamethrin [6]. The current study provides the first in vivo evidence that a crop insect pest *CSP* gene could significantly make the transgenic *Drosophila* flies overexpressing the *CSP* less susceptible, compared with control flies to insecticides with different modes of action, such as an organophosphate (omethoate), a neonicotinoid (imidacloprid, a nicotinic acetylcholine receptor blocker) and a pyrethroid (cypermethrin, a sodium channel blocker) (Figure 4). These results are supported by our *in silico* interaction results, in which AgosCSP5 was shown to form stable complex with these insecticides and have a higher affinity than with semiochemicals (Figure 5 and Table 3). 

However, the *AgosCSP5*-overexpressing transgenic flies exposed to deltamethrin and tau-fluvalinate displayed levels of susceptibility similar to that of control flies, in contrast to the results of the mosquito CSP AgamSAP2 [6]. It is not surprising that AgosCSP5 and AgamSAP2 act on different insecticides and function differently to confer insecticide resistance. They are not phylogenetically clustered together (Figure 1) and have only a 21.7% identity. Among 30 amino acid residues of AgosCSP5 predicted to participate in the binding of imidacloprid (Figure 5), only four residues (Y23, C74, C77 and Q81 of AgosCSP5) are conserved between two proteins (Supplementary Figure S3). The hydrogen bond forming residues of AgosCSP5, Arg64 in deltamethrin binding and Asp19 in imidacloprid binding, are not present in AgamSAP2. Other AgosCSPs, such as AgosCSP4 and AgosCSP6, may also participate in conferring insecticide resistance in the cotton aphids, as their transcript expressions were upregulated by insecticide treatments. It is possible that, unlike AgamSAP2, AgosCSP5 has a wider specificity and functions via antagonism of acetylcholinesterase inhibition by omethoate, nicotinic acetylcholine receptor inactivation by imidacloprid and sodium channel inactivation by cypermethrin and, thus, reduces the susceptibility of the transgenic *Drosophila* flies to these insecticides by either preventing insecticide function on the nervous system or facilitating its detoxification, possibly through sequestration.

## Figures and Tables

**Table 1:** Annotation and comparison of chemosensory proteins of the cotton aphid identified from different populations.

Name Used in This Study	NCBI ID and Name of Identical Sequence Identified in Different Populations				Genome ID (Extron Position)	Strand	Scaffold ID ^A^	Intron Size (bp)	Distance (bp) between CSPs on Same Scaffold ^C^
	Gu et al., 2013 [12]	Xu et al., 2009 [13]	Li et al., 2013 ^B^	Quan et al., 2019 [29]					
*AgosCSP1*	AGE97641.1 CSP1	n.d.	n.d.	XP_027847842.1 EBP3	NW_021009645.1 (539802...539531, 533989...533748)	Plus/Minus	AgSCF3568	5542	CSP4-CSP1-CSP6 16157, 16382
*AgosCSP2*	AGE97642.1 CSP2	n.d.	AGG38799.1 CSP2	XP_027838481.1 EBP3	NW_021007053.1 (273051...273247, 273952...274160)	Plus/Plus	AgSCF0976	705	CSP2-CSP9 248
*AgosCSP3*	n.d.	n.d.	n.d. n.d.	XP_027838481.1 EBP3 n.	NW_021007053.1 (273051...273247, 273952...274160)	Plus/Plus	AgSCF0976	705	CSP2-CSP9 248
*AgosCSP4*	AGE97643.1 CSP4	ACJ64044.1 CSP1	AGG38798.1 CSP1	XP_027847843.1 CSP	NW_021009645.1 (524668...524440, 523853...523645)	Plus/Minus	AgSCF3568	587	CSP4-CSP1-CSP6 16157, 16382
*AgosCSP5*	AGE97644.1 CSP5	ACJ64045.1 CSP2	n.d.	XP_027840827.1 OBPa10	NW_021007494.1 (888814...889061, 890414...890586)	Plus/Plus	AgSCF1417	1353	CSP8-CSP 13830
*AgosCSP6*	AGE97645.1 CSP6	n.d.	n.d.	XP_027847792.1 UCP	NW_021009645.1 (550130...549931, 548515...548313)	Plus/Minus	AgSCF3568	1416	CSP4-CSP1-CSP6 16157, 16382
*AgosCSP7*	AGE97646.1 CSP7	n.d.	AGG38800.1 CSP8	XP_027839340.1 EBP3	NW_021007172.1 (309846...309812, 303813...303527, 302266...302119)	Plus/Minus	AgSCF1095	5999, 1261	CSP10-CSP7 136286
*AgosCSP8*	AGE97647.1 CSP8	n.d.	AGG38801.1 CSP17	XP_027840825.1 EBP3	NW_021007494.1 (870401...870707, 874801...874984)	Plus/Plus	AgSCF1417	4094	CSP8-CSP5 13830
*AgosCSP9*	AGE97648.1 CSP9	n.d.	n.d.	XP_027838478.1 ATP1	NW_021007053.1 (276758...276386, 274555...274408)	Plus/Minus	AgSCF0976	1831	CSP2-CSP9 248
*AgosCSP10*	AGE97649.1 CSP10	ACJ64046.1 CSP4	n.d.	XP_027839341.1 OBPa10	NW_021007172.1 (162107...162153, 162322...162542, 165646...165833)	Plus/Plus	AgSCF1095	169, 3104	CSP10-CSP7 136286
	Na et al., 2014 ^B^	Li et al., 2016 [37]	Fan et al., 2018 [38]						
*AgosCSP11*	AHX71992.1 CSP3	n.d.	AZQ24961 AgifCSP	n.d.	n.d.				
*AgosCSP12*	AHX71993.1 CSP4	ACI03402.1 AcerCSP1	AZQ24957 AgifCSP	n.d.	n.d.				

^A^ Apollo *Aphis gossypii* isolate AGOS-L3 breed cotton aphid unplaced genomic scaffold, ASM401081v1. ^B^ no publication, direct submission in NCBI with different ID. ^C^ CSPs are located on four genome clusters (CSP1–CSP4–CSP6; CSP2–CSP9; CSP5–CSP8; CSP7–CSP10). n.d. not detected. CSP: chemosensory protein; ATP1: allergen Tha p 1-like; EBP3: ejaculatory bulb-specific protein 3-like; OBPa1: odorant-binding protein A10; UCP: uncharacterised protein; Agos: *Aphis gossypii*.

**Table 2:** Toxicity of omethoate to transgenic strain overexpressing *AgosCSP5* and control strain of *Drosophila melanogaster*.

Strains	Number ^a^	Slope ± SEM	LC50 (mg(a.i.)/L) (95% CL ^b^)	Chi-Square (Df) ^c^	Resistance Ratio ^d^
*Hsp70*–*GAL4* > *UAS*	960	2.18 ± 0.15	5.76 (4.24–7.42)	264.63 (50)	1
*Hsp70*–*GAL4* > *UAS*–*CSP5*	1005	2.83 ± 0.17	15.02 (12.74–18.06)	164.30 (50)	2.61

^a^ Total number of apterous adult aphids used in three biological bioassays. ^b^ CL, confidence interval limit. ^c^ Df, degree of freedom. ^d^ LC_50_ of the resistant strain/LC_50_ of the susceptible strain.

**Table 3:** Molecular docking scores of insecticides and semiochemicals against AgosCSP5.

Ligands	Free Binding Energy (kJ mol^−1^)
Omethoate	−20.1
Fluvalinate	−13.0
Imidacloprid	−22.6
Cypermethrin	−17.2
Deltamethrin	−18.8
Caryophyllene	−16.3
(*E*)-β-farnesene	−21.3
(*Z*)-3-hexenyl acetate	−18.4

## Data Availability

The authors confirm that the data supporting the findings of this study are available within the article and its Supplementary Materials.

## Supplementary Materials

The following are available online at https://www.mdpi.com/article/10.3390/insects12040335/s1, Supplementary Figure S1: Root–mean square deviation (RMSD) trajectory for AgosCSP5 during 50 ns of simulation. Supplementary Figure S2: Homology model of AgosCSP5 and amino acids involved in its binding site. Supplementary Figure S3: Alignment between mature AgamSAP2 and AgosCSP5. Supplemental Table S1. The primer information of *A. gossypii* and D. melanogaster for RT-qPCR. Supplementary Table S2. Toxicity of omethoate to the *A. gossypii* strain used in this study.
