# Supplementary material for: Evidence for the Involvement of the Chemosensory Protein AgosCSP5 in Resistance to Insecticides in the Cotton Aphid, Aphis gossypii
Source: Insects. 2021 Apr 9;12(4):335. doi: 10.3390/insects12040335 (PMC8070451; doi:10.3390/insects12040335)
Supplement: Supplementary file 1 [file insects-12-00335-s001.pdf]

Supplementary Figures

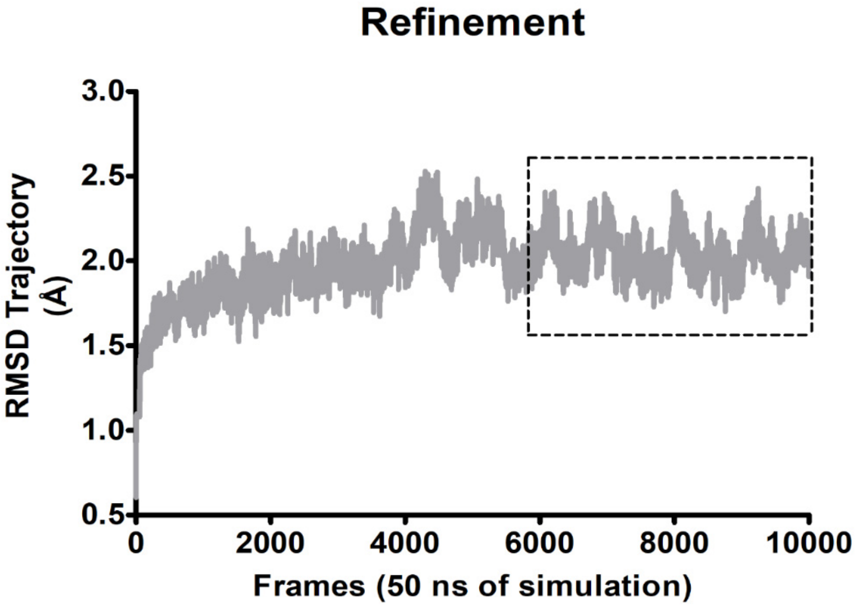

Supplementary Figure 1. Root-mean square deviation (RMSD) trajectory for AgosCSP5during 50 ns of simulation. Dotted red rectangle indicates region where representative structures were analyzed based on stereochemical quality through ProCheck server.

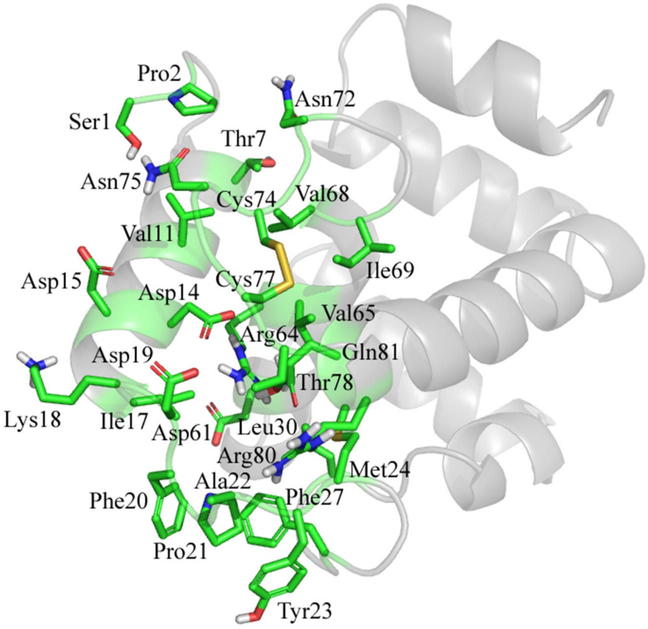

Supplementary Figure 2. Homology model of AgosCSP5 and amino acids involved in its binding site.

```
AgosCSP5      SPAGTATAAAVSADDEIKDFPAYMKRFDKLNVEQVLNNDRVLASHLKCFLNEGPCVQQSR      60
AgamSAP2      -------------------QEQYTTKYDGIDLDEILKSDRLFNNYFKCLMDEGRCTPDGN      41
                                    * .::* ::::::*:.**:: .::**:::** *. :..

AgosCSP5      DLKRVIPVIANNGCNGCTERQMTTIKKSLNFLRTKKPTEWARLVKIYDPSGTKLNKFLDA      120
AgamSAP2      ELKKILPEALQTNCEKCSEKQRSGAIKVINYVIENRKEQWDALQKKYDPENLYVEKYREE      101
              :**:::*   :..*: *:*:* :   * :*::  ::  :*  * * ***..  ::*: :

AgosCSP5      ---------   120
AgamSAP2      AKKEGIKLE   110
```

Supplementary Figure 3. Alignment between mature AgamSAP2 and AgosCSP5. The residues that participate the binding of imidacloprid and deltamethrin are highlighted in yellow, of which the conserved residues between AgosCSP5 and AgamSAP2 are highlighted in green. The residues that form a hydrogen bond with one of the nitrogen-bound oxygens of imidacloprid (D19) and deltamethrin (R64) are highlighted in red.

Supplementary Tables

Supplemental Table 1. The primer information of *A. gossypii* and *D. melanogaster* for RT-qPCR

Primer name	RT-qPCR Primer sequences (5'-3')	References
AgosCSP1	Forward: GTGCGACACTCAAGTCAAGC<br>Reverse: TGACGACGTTTCTCTGATGG	Gu et al., 2013 [19]
AgosCSP2	Forward: TTCAGCAGCGGAAGAAAAGT<br>Reverse: TTTCCTTCGTCAAGCAAACA	Gu et al., 2013 [19]
AgosCSP4	Forward: GCCACAAAAAGATGCCGTAG<br>Reverse: TTGTCCAACAGACATTGAACG	Gu et al., 2013 [19]
AgosCSP5	Forward: GACAAGCTCAACGTCGAACA<br>Reverse: TCTTGACGAGTCTTGCCCAT	Gu et al., 2013 [19]
AgosCSP6	Forward: GACAGCCCCGGCTAAATATAC<br>Reverse: TTTTCCCGTTTCCATCAGAC	Gu et al., 2013 [19]
AgosCSP7	Forward: ATCGTCGTCAAGTGTCACCA<br>Reverse: TGCCGCTAAGGTTTCAGATT	Gu et al., 2013 [19]
AgosCSP8	Forward: CGCTGTTAGCCGTTACGATT<br>Reverse: AACATCATCGTCTGCTGCTG	Gu et al., 2013 [19]
AgosCSP9	Forward: GTCAGCGTTTTGCCTGAACT<br>Reverse: TCGTCGGTATCCCTTTTGAC	Gu et al., 2013 [19]
AgosCSP10	Forward: CGTAGGCGTTTCAATCTCCG<br>Reverse: GTGCACGGTCCTTCATTCAG	Gu et al., 2013 [19]
Drosophila reference gene *Rpl32*	Forward: GCGCTTGTTCGATCCGTAAC<br>Reverse: GCCCAAGGGTATCGACAACA	Wang et al., 2020 [40]
Cotton aphid reference gene *EF1-α* EU019874.1	Forward GAAGCCTGGTATGGTTGTCGT<br>Reverse GGGTGGGTTGTTCTTTGTG	Ma et al., 2016 [41]

Supplementary Table 2. Toxicity of omethoate to the *A. gossypii* strain used in this study.

Number [a]	LC_{10} (mg/L) (95% CL[b])	LC_{50} (mg/L) (95% CL[b])	LC_{90} (mg/L) (95% CL[b])	Slope±SEM	Chi-square	Df[d]
2776	574.398<br>(477.926-653.816)<br>0.192±0.080[c]	1029.108<br>(956.254-1094.540)<br>0.611±0.202[c]	1843.777<br>(1691.617-2072.485)<br>0.853±0.041[c]	5.060±0.246	122.62	34

[a] Total number of apterous adult aphids used in three biological bioassays. The cotton aphids were collected two years ago from a cotton field. [b] CL, confidence interval limit. [c] The results are shown as mortality ± SEM at each concentration. [d] Df, degree of freedom.
